# Near-surface coherent structures explored by large eddy simulation of entire tropical cyclones

**DOI:** 10.1038/s41598-017-03848-w

**Published:** 2017-06-19

**Authors:** Junshi Ito, Tsutao Oizumi, Hiroshi Niino

**Affiliations:** 1 Meteorological Research Insititute, Tsukuba, Ibaraki Japan; 2 Atmosphere and Ocean Research Institute, The University of Tokyo, Kashiwa, Chiba Japan; 3 Japan Agency for Marine-Earth Science and Technology, Yokohama, Kanagawa Japan

## Abstract

Taking advantage of the huge computational power of a massive parallel supercomputer (K-supercomputer), this study conducts large eddy simulations of entire tropical cyclones by employing a numerical weather prediction model, and explores near-surface coherent structures. The maximum of the near-surface wind changes little from that simulated based on coarse-resolution runs. Three kinds of coherent structures appeared inside the boundary layer. The first is a Type-A roll, which is caused by an inflection-point instability of the radial flow and prevails outside the radius of maximum wind. The second is a Type-B roll that also appears to be caused by an inflection-point instability but of both radial and tangential winds. Its roll axis is almost orthogonal to the Type-A roll. The third is a Type-C roll, which occurs inside the radius of maximum wind and only near the surface. It transports horizontal momentum in an up-gradient sense and causes the largest gusts.

## Introduction

Tropical cyclones (TCs) are among the most destructive atmospheric disturbances and cause large-scale disasters. This study focuses on organized structures in the lowest layer of the TCs near the surface. This portion of the atmosphere, called the TC boundary layer, is characterized by vigorous turbulence due to the presence of the surface friction. Because of extraordinarily strong vertical shear which changes direction with height^1^, the TC boundary layer exhibits very different characteristics from those of the usual atmospheric boundary layer^2^.

Although strong winds associated with TCs occur over a few hundred km from the TC centre, damage surveys after a TC passage often reveal sub-kilometre-scale damage swaths that are considered to be footprints of localized gusts^3^. Doppler-radar observations of a TC boundary layer revealed existence of horizontal roll structures which may explain the sub-kilometer-scale wind variations as found in the damage survey^4^. Such a characteristic pattern is considered to result from some instabilities that are intrinsic to the TC boundary layer.

A previous study^4^ speculated that roll structures are related to thermal convection in a vertical wind shear^5^, but analytical^6, 7^ and numerical^8, 9^ studies indicated that they are likely to be caused by a dynamic instability of the boundary-layer flow associated with the vertical shear, similar to that of the Ekman-layer flow. Since the horizontal scales of some roll structures are hundreds of times smaller than the scale of the TC itself, previous analytical and numerical studies used a number of simplifications. Thus, uncertainties remain with regard to the structures in the TC boundary layer^10^.

Meteorological agencies routinely run their numerical weather prediction (NWP) models, which numerically integrate, with respect to time, the governing equations describing a variety of atmospheric physical processes^11^. NWP models are also used to predict TC’s strength and path, but the horizontal resolution is typically limited to several kilometres. Thus, small-scale structures and turbulence in the TC boundary layer are not resolved and their effects must be somehow expressed in terms of resolved-scale variables. This is called turbulence parameterization. Since the small-scale structures (including rolls) in the boundary layer are poorly understood, turbulence parameterization of the TC boundary layer has been extensively studied^12, 13^. It is known that the TC-scale circulation is significantly affected by this parameterization^14–17^, since turbulence plays an important role in transferring momentum, heat and moisture from the surface into the atmosphere^18^.

The most reliable way to investigate small-scale structures in the TC boundary layer, without relying on approximations, would be to perform a simple numerical simulation of an entire TC while resolving the small-scale structures. Several studies have investigated fine-scale structures in the TC boundary layer using a large eddy simulation (LES)^8, 13, 19, 20^, but none have simulated an entire TC with homogeneous horizontal grids because of limited computational resources. The K computer, Japan’s most powerful supercomputer^21^, enables us to perform such extraordinarily large computations with grid numbers exceeding 10^10^. The hundreds of thousands of homogeneous processing cores of the K computer allow us to conduct massive parallel computations (e.g., global NWP models)^22, 23^.

The simulation model used for the present study is a LES version of the Japan Meteorological Agency’s operational regional weather prediction model, which is based on a fully compressible, non-hydrostatic model (JMA-NHM)^24^. The horizontal grid spacing adopted in this study is 100 m. This resolution is reasonably fine for the purpose of resolving large eddies in the TC boundary layer, since roll structures in the boundary layer investigated in a previous LES study^8^ varied little between horizontal grid spacings of 120 and 70 m (Dr. Nakanishi, private communication). For our present simulation we do not need to worry about complicated effects of the horizontal boundaries between two adjacent regions having different resolutions, and the simulation results can be used to study boundary-layer structures affected by radial variation of a TC^10^.

To save resources, we first undertook a preliminary run (P run) with a horizontal resolution *dx* of 2 km to generate a mature TC. The results of the P run after five days of integration are interpolated to provide the initial conditions for the LES run, which has the same domain size as the P run.

## Results

Two cases for the initial disturbances having different sizes and sea-surface temperatures (SSTs) are examined (see Method section for details): one is a moderate TC (MTC) with a central surface pressure *P*_s_ of 950 hPa, while the other is a strong TC (STC) where *P*_s_ reaches 920 hPa after 120 hours of the P run integration. Since the results of the two cases exhibit qualitatively similar characteristics as for small-scale structures, we will mostly present results for the MTC, unless stated otherwise. The results of the LES run to be discussed below are for 10 hours after its initiation (i.e., 130 hours after initiation of the P run). The origin of the horizontal cross-sections is taken as the TC centre, which is determined as the point giving the best axisymmetry of the surface pressure.

**Figure 1:**
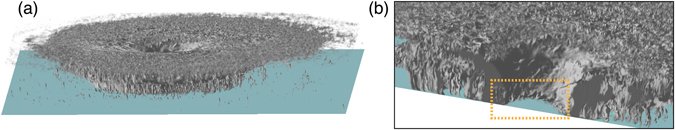
Three-dimensional view of simulated cloud amount visualized by volume rendering. (**a)** the central 200 km × 200 km region of the computation domain of the MTC. (**b)** Close-up view of a cross-section of the TC core. The region with the cloud features associated with Type-C rolls is indicated by the orange rectangle.

Figure 1 shows the three-dimensional view of the simulated cloud water and ice mixing ratio, *q*_w_, in the LES run. In addition to the fundamental components of a TC, such as an eyewall and an upper-level outflow, a number of fine-scale structures are seen. The supplementary movie shows the time evolution of the simulated cloud water between 9 and 10 hours after the start of the LES run. One can see growth and decay of cumulus clouds, which are important elements affecting the TC-scale circulation.

### TC-scale structures

**Figure 2:**
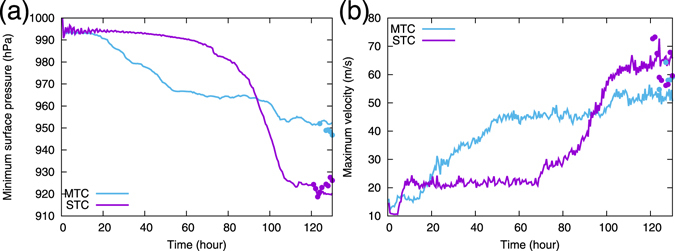
Time series of TC intensities for MTC and STC: (**a)** minimum surface pressure and (**b)** maximum surface wind for P runs (solid lines) and for LES runs started from *t* *=* 120 hours (dots). These plots are based on outputs at every 30 minutes for P runs, and those at every 1 hour after 120 hours for LES runs except that no outputs were made at *t* = 120–123 and 125–127 hours for LES run of MTC, and every 1 hour after *t* = 120 hours for LES run of STC.

**Figure 3:**
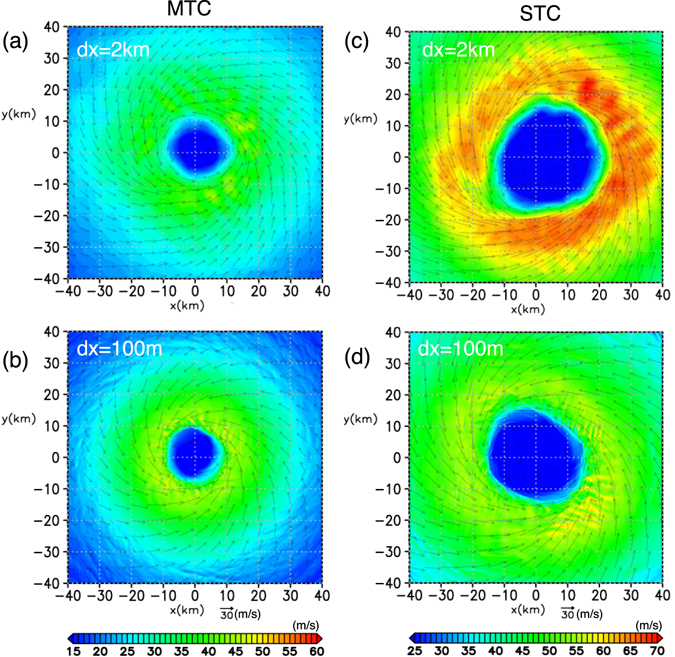
Surface wind speed: Horizontal cross-sections at *z* = 10 m for *t* = 130 hr are shown. (**a)** P run for MTC; (**b)** LES run for MTC; (**c)** P run for STC; (**d)** LES run for STC. Note that the colour scales are different between MTC and STC.

A comparison between the P and LES runs shows that the magnitudes of the minimum surface pressure and maximum of the surface winds (defined by horizontal wind speeds at the height of *z* = 10 m) in the LES run are nearly the same as those in the P run for MTC, but are slightly weaker than those in the P run for STC (Figs 2 and 3). In these experiments with 10 hours of time integration, the increase in resolution from the P run to the LES run does not seem to cause a noticeable intensification of the TCs. This contrasts with the previous LES studies in which the maximum surface wind increased when LES is nested in a coarse resolution model^19^.

A remarkable difference between the P and LES runs for MTC is that the eyewall radius shrinks in the LES run (Fig. 3 and Supplementary Fig. S1). The structural differences between the P and LES runs are evident in the radius–height cross-sections (Supplementary Fig. S2). The radius of the maximum tangential wind, *u*_t_ (Supplementary Fig. S2b), and that of the eyewall cloud (Supplementary Fig. S2c) are apparently smaller in the LES run. Stronger radial inflow near the surface occurs in the LES run (Supplementary Fig. S2a). In the caption of Supplementary Figs S1 and S2 and hereafter, *u*_*r*_ and *u*_*t*_ are the horizontal velocity components in radial and tangential directions, respectively, *w* is the vertical velocity component; overbars and primes represent azimuthal average and deviation from the average, respectively.

The P runs and LES runs also exhibit differences in the parameterized eddy viscosity^25^ whose magnitudes depend on horizontal grid size *dx*. Although resolved vertical momentum flux is largely contributed by the roll structures in the LES run, the absolute value of sub-grid scale vertical momentum flux is reduced and that of total flux is smaller than that in the P run (Supplementary Figs S2d and S3). The TC boundary layer (inflow layer) height is also decreased in the LES run (Supplementary Fig. S4a). These changes possibly due to the difference in the eddy viscosity are consistent with previous studies^26^, and might affect the change in the eyewall radius. The heights of the boundary layer and the maximum tangential wind (301 m and 275 m at *r* = 15 km, respectively) in the LES run are somewhat lower than those estimated by dropsonde observations over many TCs, which is higher than 500 m^27, 28^.

The decrease of TC boundary layer height is more significant in STC (Supplementary Fig. S4b). However, a similar reduction of the eyewall radius does not occur and the surface winds are weaker for the LES run for STC (Fig. 3c and d). Currently, physical reason for these differences are unclear.

### Small-scale coherent structures

**Figure 4:**
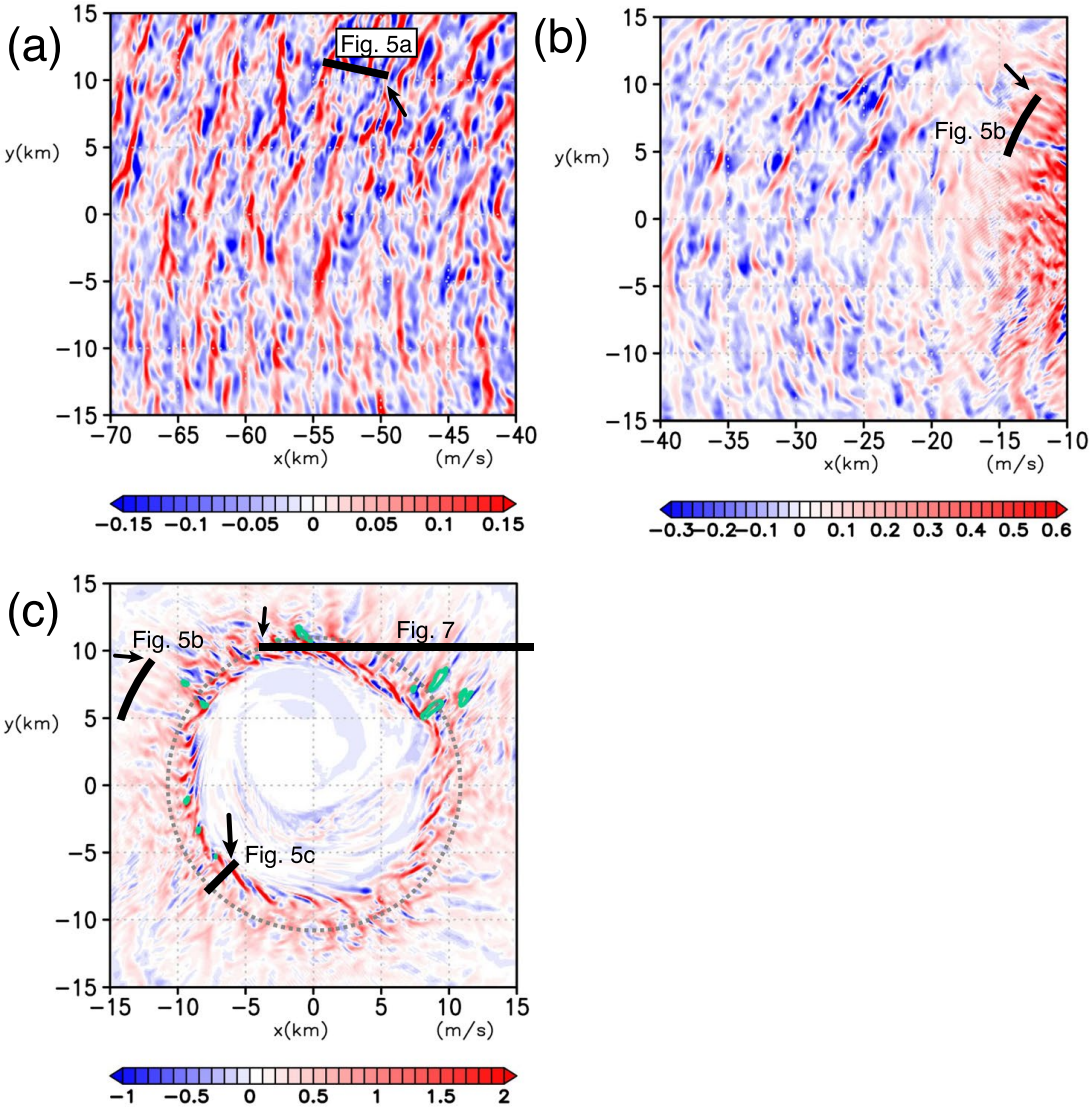
Roll structures near the surface as seen from vertical velocities in horizontal cross-sections at z = 27 m for the MTC. (**a)** Regions more distant to, (**b)** closer to, and **c** near the TC centre. Green contours in panel **c** show the locations where the horizontal wind speed at z = 10 m is greater than 55 m s^−1^; the dotted line indicates the RMW; the solid lines indicate the position where the vertical cross-sections are taken in Figs 5 and 7, and each arrow indicates the left end of the corresponding cross-section in Figs 5 and 7. Note that the colour scales differ among panels.

The LES run (Fig. 3b and d) shows that the locally strong surface winds are apparently associated with small-scale structures, which are much better resolved here than in the P run. These structures are more clearly seen in the vertical velocity, which exhibits less radial variations than those associated with the horizontal winds (Fig. 4). There exist three different types of rolls, which are characterized with linear pairs of updraft and downdraft, at different radii.

**Figure 5:**
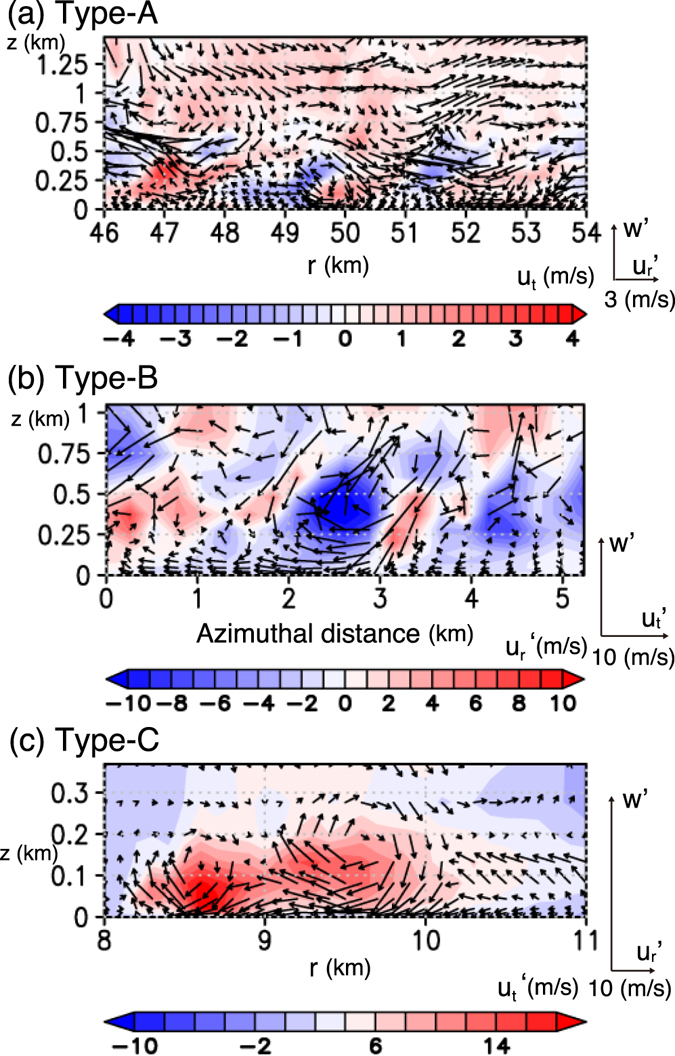
Vertical cross-sections across each type of the roll. (**a)** Radial–height cross section for the Type-A rolls (${u}_{r}^{^{\prime} },w^{\prime}$) (vectors) and ${u}_{t}^{^{\prime} }$ (shading), (**b)** azimuthal–height cross section for the Type-B rolls (${u}_{t}^{^{\prime} },w^{\prime}$) (vectors) and ${u}_{r}^{^{\prime} }$ (shading), and **c** radial–height cross section for the Type-C rolls (${u}_{r}^{^{\prime} },w^{\prime}$) (vectors) and ${u}_{t}^{^{\prime} }$ (shading). The position of each vertical cross-section is shown in Fig. 4; the left end of each cross-section is shown by the arrow. Note that the colour scale and the size of the cross sections are different for each panel.

Type-A rolls prevail outside the radius of the maximum wind (RMW) and have their axes oriented nearly parallel to the tangential wind (Fig. 4a and b). Type-B rolls are found near the RMW at about *r* ~ 15 km (Fig. 4b and c). Their axes are nearly oriented to the radial direction and is almost orthogonal to those of Type-A rolls. Type-C rolls are found inside the RMW (8 < *r *< 10 km; Fig. 4c) and have their axes oriented nearly parallel to the tangential wind with a slight outward deflection. Near *x* ~ 10 km, Type-B and -C rolls intersect almost orthogonally. Figure 5a and c show radial–height cross sections for Type-A and -C rolls, respectively, and Fig. 5b shows an azimuthal–height cross section across Type-B rolls. To the authors’ knowledge, existence of Type-B and -C rolls has not been reported previously.

**Figure 6:**
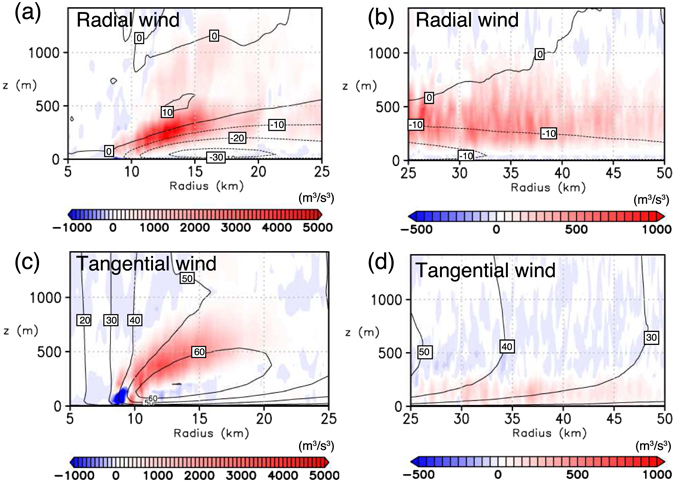
Vertical shear production of TKE in radius-height planes for the MTC: (**a)** production due to vertical shear of the radial wind $-r\cdot \overline{{u}_{r}^{^{\prime} }w^{\prime} }\frac{\partial \overline{{u}_{r}}}{\partial \,\overline{z}}$ (shading) and *u*_*r*_ (contours) for inner radii, (**b)** that for outer radii, (**c)** production due to vertical shear of the tangential wind $-r\cdot \overline{{u}_{t}^{^{\prime} }w^{\prime} }\frac{\partial \overline{{u}_{t}}}{\partial \overline{z}}$ (shading) and *u*_*t*_ (contours) for inner radii, and (**d)** that for outer radii. Note that the colour scales are different in panels.

Type-A rolls are similar to those reported in previous idealized LES studies^8, 9^. The rolls are caused by an inflection-point instability, which appears to be an analog of that in the turbulent Ekman layer^29^. Indeed, the vertical profile of the radial wind *u*_*r*_ where Type-A rolls prevail has an inflection point (${\partial }^{2}{u}_{{\rm{r}}}/\partial {z}^{2}\mathrm{=0}$), and production of turbulent kinetic energy (TKE) due to vertical shear of the radial wind is large (Fig. 6b). In the cross section shown in Fig. 5a, stronger tangential winds occur below *z* = 250 m with a horizontal spacing of 2–3 km. They appear to be caused by the downdrafts associated with Type-A rolls (e.g. at *r* ~ 48 km, 50 km, and 52 km). These roll structures are not as clear as those demonstrated in other LESs^8, 9, 13^ owing to more complex configurations including moist processes and radial inhomogeneties.

**Figure 7:**
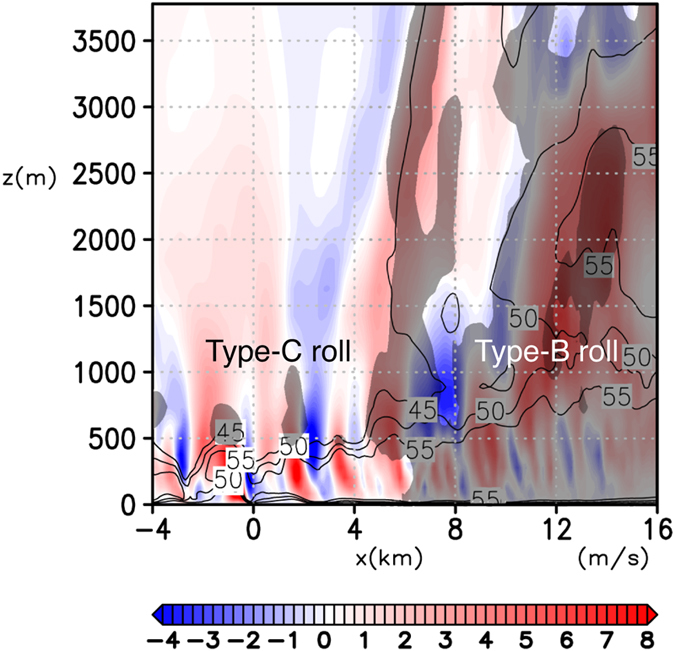
Vertical cross section of Type-B and C rolls. Vertical velocity (color shading), horizontal wind speed in the unit of m/s (contours), and cloud water mixing ratio *q*_*W*_ (grey shading; thin grey denote *q*_*W*_ > 1 g/kg and thick grey *q*_*W*_ > 3 g/kg) in vertical (*x*-z) cross section near the RMW (*Y* = 10.5 km in Fig. 4c) for the MTC.

Type-B rolls prevail near the maxima of the tangential wind where vertical profiles of the horizontal winds are apparently different from those in the outer radii for which Type-A rolls prevail (Figs 6 and Supplementary S5). Unlike Type-A rolls, instabilities due to inflection points of both radial and tangential wind whose profiles are characteristic of TCs appear to cause Type-B rolls. The updrafts associated with Type-B rolls are connected to those in eyewall clouds aloft (Fig. 7).

Type-C rolls have their circulation center at around z = 100–150 m (Fig. 6c). While an inflection point of the tangential wind *u*_*t*_ is located at *z* = 240 m and inflection points of the radial wind ur are located at *z* = 120 m and 450 m (Fig. Supplementary S5), TKE production due to the vertical shear of *u*_*t*_ is dominant over that due to the vertical shear of radial wind and is largest right near the surface (Fig. 6c). Thus Type-C rolls does not seem to be caused by inflection point instabilities of vertical shear flow. Furthermore, the down-shear inclination of roll axes in the horizontal plane suggests that they are not caused by inflection point instabilities of horizontal shear flows either. One of the possible mechanisms is a “parallel instability” whose presence in the TC boundary layer is theoretically predicted^6^: a parallel instability of the Ekman layer with molecular viscosity was predicted theoretically^29^ and was confirmed experimentally^30, 31^, but has not been found to occur in the atmosphere^32^. The parallel instability of the Ekman layer occurs when the background rotation is very strong, and may lead to the generation of roll structures aligned nearly orthogonally to those caused by the inflection-point instability of the tangential wind. The presence of a parallel instability in the TC boundary layer may be possible only near the RMW, where the centrifugal force is large to make the “effective background rotation” significant. The horizontal spacing of simulated Type-C rolls along the azimuthal direction is ~2 km (Figs 4c and 7) and is larger than that of Type-B roll (~1 km; Figs 4c and 5b).

The surface winds have large local maxima in the regions where Type-B and C rolls prevail (Fig. 4c). The horizontal wind speed increases with height below 50 m (Supplementary Fig. S5), so that these local maxima appear to be associated with momentum transport due to downdrafts.

Unlike those of Type-B rolls, however, the updrafts of Type-C rolls do not seem to be connected to the mid-level updrafts in the eyewall clouds (Fig. 7). They are accompanied by only shallow clouds that look like ripples of the eyewall cloud near the core (Fig. 1b).

## Discussion

The present study examined small-scale coherent structures in the TC boundary layer over a wide range of radius for the first time. Three distinct rolls have been found. Type-A rolls prevail outside the RMW, where their possible impact on TC intensity has been suggested by previous studies^33^. Thus, a NWP model with a coarse resolution has to take into account such an upscale impact of the TC boundary layer to achieve better forecasts.

An examination of TKE production (Supplementary Fig. S6) as a function of radius exhibits a curious character of the Type-C rolls. Outside the RMW, where Type-A and -B rolls prevail, the turbulent energy is predominantly produced mainly by vertical shear and little by buoyancy, which is consistent with inflection-point instabilities. Near the RMW where Type-C rolls prevail, on the other hand, the vertical-shear production contributes to increasing the TKE while horizontal-shear production is significantly negative. In fact, the down-shear inclination of the Type-C rolls in the horizontal plane is opposite to that expected for a horizontal shear instability which tends to weaken the shear (Fig. 4c). The Type-C rolls appear to transport horizontal momentum in the up-gradient direction. The RMW is located where the horizontal shear production changes sign. A previous study using an axisymmetric model showed that the turbulence parameterization has considerable impacts on the maximum tangential wind^26^: the maximum velocity decreases monotonically with increasing turbulent intensity, suggesting a down-gradient transport of horizontal momentum. However, the present LES study suggests that some kind of small-scale structures such as Type-C rolls could cause an up-gradient transport of horizontal momentum. This may contribute to the larger tangential wind at *z*~200 m in the LES run than that in the P run (Supplementary Fig. S2b), though the difference in the tangential wind between the two runs is insignificant near the surface (Fig. 3).

**Figure 8:**
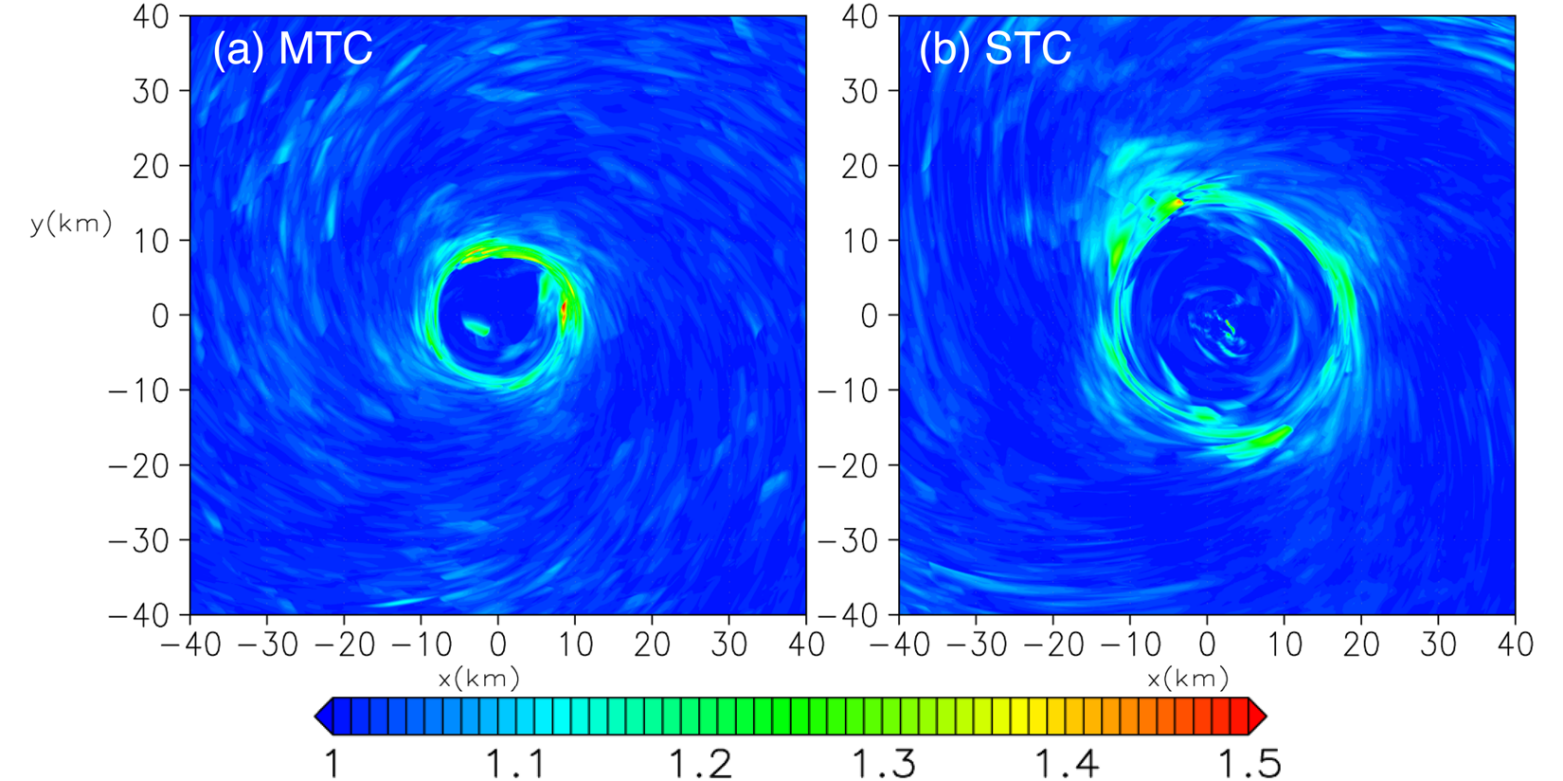
Gust factor, defined as the 3-second mean surface wind speed maximum (*U*_s3_) divided by that of the 60-second mean surface wind speed (*U*_s60_) at *z* = 10 m near the TC centre for **a** the MTC and **b** the STC.

The small-scale spatial variations of winds associated with the rolls also cause large rapid temporal variations of the wind speed at a fixed point. In wind engineering, the gust factor (which is a measure for the intensity of short-term strong winds) is important for wind-resistant design. A typical gust factor, defined by the maximum of 3-second mean surface wind divided by 60-second mean wind speed, *G*_3s_/*U*_60s_, is examined here. The World Meteorological Organization’s guideline^34^ suggests that, for a TC over an ocean, this ratio is about 1.1. We can explicitly evaluate the gust factor based on the LES run. Figure 8a and b show *G*_3s_/*U*_60s_ for the MTC and STC, respectively. The gust factor remains about 1.1 for most part of the TC, but it reaches 1.5 near the RMW due to the large velocity variance (~20 m s^−1^ at the maximum) possibly caused by Type-C rolls (Fig. 5c).

Previous studies reported monotonic increases in the maximum tangential wind with improving horizontal resolution^14, 19^. For nested models with the innermost domain having a very fine resolution, small-scale structures near the RMW that accompany large surface wind speeds exceeding 100 m s^−1^ have been reported^10, 19^. For nested models, the structure of the TC in the inner fine-resolution domain is strongly forced by that in the outer coarse-resolution domain. In the present LES run, however, all the structure of the TC is determined solely through internal dynamics. We speculate that the differences in the sensitivity of maximum wind speed to resolution are caused by theses model differences.

The LES results may be used for improving the sub-grid parameterization of the TC boundary layer. We have seen that Type-C rolls transport horizontal momentum in the radially up-gradient direction, though a parameterization of such a process is intrinsically difficult. Using the huge data of the present LES, we plan to conduct further analysis on the roll dynamics and disturbances in the eyewall cloud above the boundary layer. A more complete analysis of the TKE budget including advection, dissipation, and other terms will clarify the impact of rolls on overall TC dynamics and will contribute to a better parameterization of the TC boundary layer.

Although the net computational time needed for the present LES run is only 4 days (see Method), the actual turn-around time was about a half year which includes queuing for job executions, data transfer, and post analyses, because quite a number of people were using the K-computer system. Nevertheless, we plan to conduct additional LES runs for different idealized environments with including environmental vertical wind shear and also real cases. Development of post-K system would accelerate these lines of research.

Our approach here attempts to bypass the “Terra Incognita” or grey-zone problem where neither conventional turbulence parameterization nor the LES approach are applicable^10, 35^. While the present LES run was able to reproduce the small-scale structures, a considerable fraction of turbulent transports are still contributed by sub-grid scale motions within lower TC boundary layer (Supplementary Fig. S3). To verify that the sub-grid scale turbulence does not drastically change the characteristics of the TC boundary layer, it is desirable to perform a LES with finer horizontal and vertical resolution. On the other hand, the changes of eyewall radius and surface winds from the P runs to the LES runs were not systematic between MTC and STC. Since the simulated TCs in the LES run exhibit considerable time variation even at a mature stage, we may also need to make either a longer time integration or ensemble runs to resolve this issue. Such extremely large calculations would become possible when the post-K supercomputer is installed.

## Method

This study employs the JMA-NHM^24^, which is used for daily operational regional weather predictions at the JMA and also for research including idealized numerical experiments of TCs, extratropical cyclones and polar lows^36–39^. The computational domain covers 2000×2000 km^2^ and 23 km in the horizontal and vertical directions, respectively. Horizontal boundary conditions are doubly periodic. A three-ice single-moment bulk scheme^40^ is used for parameterization of cloud microphysics. The turbulence parameterization employs Deardorff’s scheme for both P runs and LES runs based on simple down-gradient transport using eddy viscosity^25^ with the closure coefficient of 0.1. Long-wave and short-wave radiation are included^24^. The calculation is performed on an *f* plane at 10° North. The settings adopted for the P and LES runs are described below.

### Configuration of the P run

In the P runs, a horizontal grid size of d *x* = 2 km is used. The grid numbers in the horizontal directions are both 1000. There are 60 model levels in the vertical direction, where grid spacing *dz* increases from 10 m near the surface to 818 m near the top of the calculation domain; *dz* is less than 100 m below *z* = 538 m, and is less than 200 m below *z* = 1522 m. A sponge layer is placed above 17 km to suppress reflections of gravity waves from the top boundary.

The initial environment is horizontally uniform and is given by typical sounding data in the tropics during the “hurricane” season^41^. No environmental wind is imposed. The initial disturbances are given by a vortex of an analytic form^42^ with a maximum wind speed of 15 m s^−1^. Two different cases are examined: (1) the RMW of the initial vortex is 50 km and the SST is 300 K (MTC), and (2) the RMW of the initial vortex is 300 km and the SST is 303 K (STC). Time integrations are performed for 130 hr, with a time step d*t* = 8s. A TC develops spontaneously from the initial vortex in each case (Fig. 2). The grid-point values at 120 hr after initiation are interpolated to prepare the initial values for the subsequent LES run.

### Configuration of the LES run

The LES run uses the same domain size as the P run but has a horizontal grid spacing d *x* = 100 m everywhere. The horizontal grid number is 20,000 × 20,000, while the vertical grid number and the sponge layer are the same as those of the P run. A time step of 0.8 s is used, and time integration is 10 hours for both the MTC and the STC. Thus, the LES run requires nearly 4,000 times the computing resources required by the P run.

The LES run was conducted using 9,216 nodes (each node has 8 processing units and 16 GB of memory) of the K computer, which correspond to almost 1/8 of the total system resources. A time integration of 1 hour takes an execution time of approximately 9.5 hours, excluding the time required for disk input and output. Storage of the output data was also problematic: the file size of a temporal output to restart the time integration reached approximately 17 TB.

### Code availability

JMA-NHM is available under collaborative framework between Meteorological Research Institute and related institutes or universities. The output from model simulations used here and post processing code are available on request.

## Electronic supplementary material

Supplementary Video

Supplementary Information
