# Supplementary material for: Near-surface coherent structures explored by large eddy simulation of entire tropical cyclones
Source: Sci Rep. 2017 Jun 19;7:3798. doi: 10.1038/s41598-017-03848-w (PMC5476618; doi:10.1038/s41598-017-03848-w)
Supplement: Supplementary file 2 — Supplementary Information [file 41598_2017_3848_MOESM2_ESM.pdf]

Near-surface coherent structures explored by large eddy simulation of entire tropical cyclones

Supplementary Information

Junshi Ito, Tsutao Oizumi, and Hiroshi Niino

Radial profiles of tangential velocity

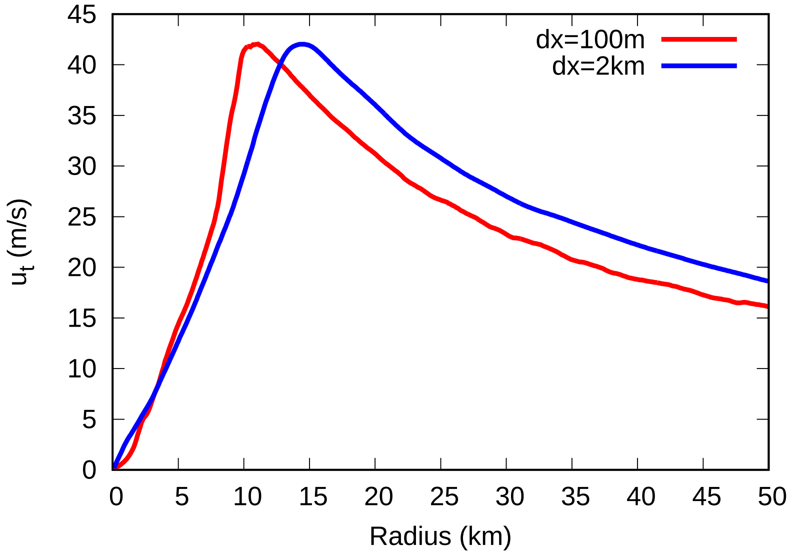

Figure S1: Tangential velocity u_t at the surface (z=10 m). Radial profiles of u_t for the P run and the LES run for MTC at t =130 hours.

Difference between P and LES runs

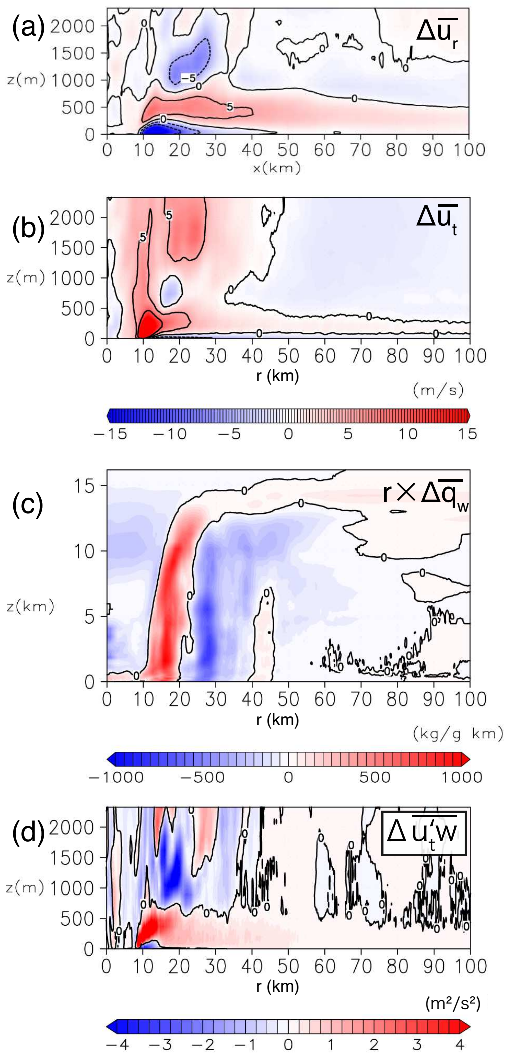

Figure S2: Differences between P and LES runs. Azimuthally averaged cross-sections in the sense of the LES run minus P run at $t = 130$ hr for MTC are shown. The individual panels show the differences in **a**: the radial wind, $\Delta\overline{u_\mathrm{r}}$; **b**: the tangential wind, $\Delta\overline{u_\mathrm{t}}$; **c**: the cloud water amount multiplied by the radius, $r \times \Delta\overline{q_\mathrm{w}}$; and **d**: the vertical turbulent flux of the tangential wind $\Delta\overline{u_\mathrm{t}' w'}$ including the contributions of sub-grid turbulence.

Resolved vs. Sub-grid vertical momentum flux

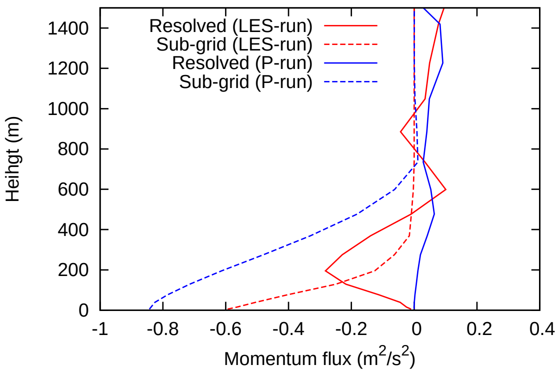

Figure S3: Resolved and sub-grid vertical flux of azimuthal momentum $\overline{u_t' w'}$ at $r = 40$ km at $t = 130$ hours. Vertical profiles of the resolved and sub-grid fluxes are shown by solid and dotted lines, respectively, where those for the LES run and P run are shown by red and blue colours, respectively.

TC boundary layer heights

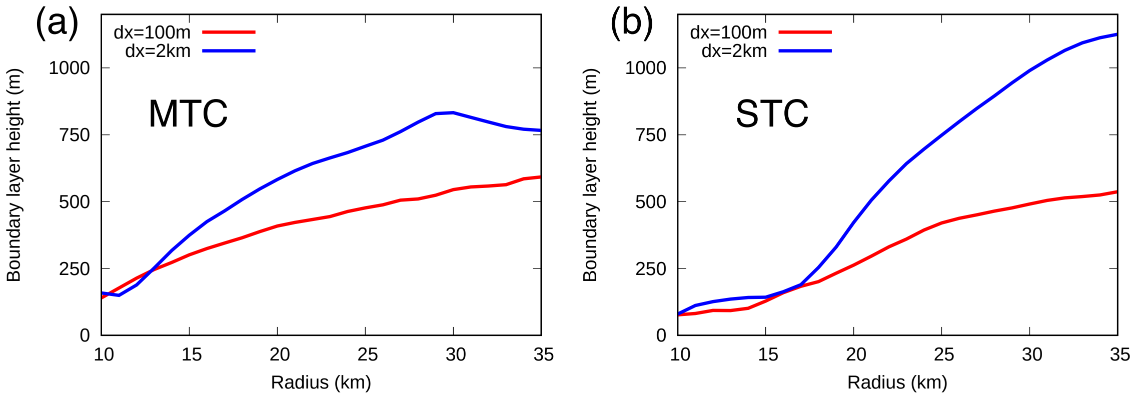

Figure S4: Radial distribution of TC boundary layer heights for the P run and LES run for (a) MTC and (b) STC at t =130 hours. Note that the TC boundary layer height is defined as the height at which the inflow decreases to 10 % of its largest azimuthal-mean value.

Vertical profiles of radial and tangential velocities

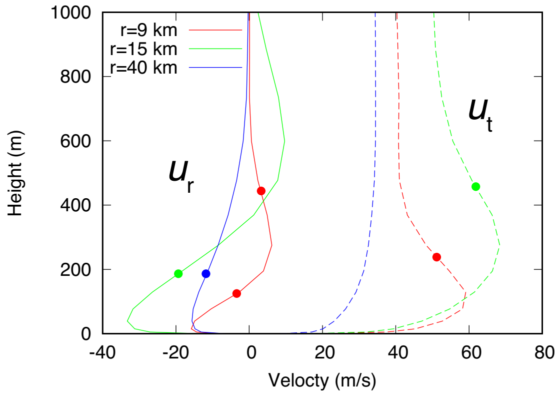

Figure S5: Vertical profiles of radial (solid lines) and tangential (dotted lines) velocities,u_r and u_t, at the radius r of 9, 15, and 40 km for MTC at t =130 hours. Solid circles indicate the heights of inflection points ($\partial^2 u_r/\partial z^2 = 0$ and $\partial^2 u_t/\partial z^2 = 0$) for each radius.

TKE budget equation and TKE production

The TKE budget equation is written as

$$\frac{1}{2}\frac{\partial\overline{u_i'u_i'}}{\partial t} = -\overline{u_i'u_j'}\frac{\partial\overline{u}_i}{\partial x_j} + \overline{\frac{g}{\theta_0}w'\theta'} + \cdots, \tag{1}$$

$$= -\Big(\overline{u_r'^2}\frac{\partial\overline{u_r}}{\partial r} + \overline{u_r'u_t'}\frac{1}{r}\frac{\partial\overline{u_r}}{\partial\theta} + \overline{u_r'u_t'}\frac{\partial\overline{u_t}}{\partial r} + \overline{u_t'^2}\frac{1}{r}\frac{\partial\overline{u_t}}{\partial\theta}\Big) \tag{2}$$

$$-\Big(\overline{u_r'w'}\frac{\partial\overline{u_r}}{\partial z} + \overline{u_t'w'}\frac{\partial\overline{u_t}}{\partial z})$$

$$+\overline{\frac{g}{\theta_0}w'\theta_v'} + \cdots,$$

where g is the gravitational constant, θ_v is the virtual potential temperature which is found to differ little from θ, and θ_0 is the standard temperature $\equiv$ 300 K. In the right hand side of Eq. (2), the first line represents TKE production by horizontal shear, the second line represents that by vertical shear, and the third line represents that by buoyancy. The terms other than production (i.e. advection, turbulent transport, pressure transport, Coriolis, and cetrifugal terms which redistribute TKE among velocity components, and dissipation and other shear production terms) are not shown, since they do not contribute to increase the net TKE.

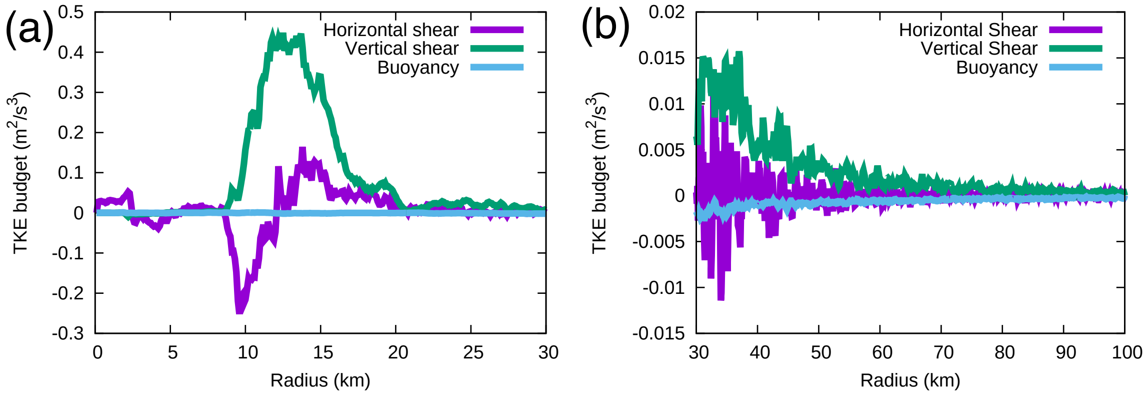

Figure S6: Radial distributions of production terms in TKE budget: productions by horizontal shear (purple), vertical shear (green), and buoyancy (light blue) are shown for MTC at $z = 195$ m at $t = 130$ hours. Panel **a** shows radial distribution for $0 < r < 30$ km and **b** for $30 < r < 100$ km, respectively. Notice that vertical scales in the ordinate are different between these panels.

Supplementary movie

Time evolution of the simulated cloud water amount. The central region of the computational domain of the MTC from t=129 to 130 hr is shown.
